# Effects of green coffee aqueous extract supplementation on glycemic indices, lipid profile, CRP, and malondialdehyde in patients with type 2 diabetes: a randomized, double-blind, placebo-controlled trial

**DOI:** 10.3389/fnut.2023.1241844

**Published:** 2023-11-16

**Authors:** Sajad Khalili-Moghadam, Mehdi Hedayati, Mahdieh Golzarand, Parvin Mirmiran

**Affiliations:** ^1^Department of Clinical Nutrition and Dietetics, Faculty of Nutrition and Food Technology, National Nutrition and Food Technology, Research Institute, Shahid Beheshti University of Medical Sciences, Tehran, Iran; ^2^Cellular and Molecular Endocrine Research Center, Research Institute for Endocrine Sciences, Shahid Beheshti University of Medical Sciences, Tehran, Iran; ^3^Nutrition and Endocrine Research Center, Research Institute for Endocrine Sciences, Shahid Beheshti University of Medical Sciences, Tehran, Iran

**Keywords:** type 2 diabetes, Hs-CRP, Malondialdehyde, Insulin, lipid profile, green coffee

## Abstract

**Background/objectives:**

Studies have reported the health benefits of green coffee extract (GCE) in experimental models. In the current study, we aimed to determine whether supplementation with GCE improves glycemic indices, inflammation, and oxidative stress in patients with type 2 diabetes (T2D).

**Methods and study design:**

This randomized, double-blind, placebo-controlled trial included 44 patients (26 male and 18 female) with T2D and overweight/obesity. After blocked randomization, patients received either capsules containing 400 mg GCE twice per day (*n* = 22) or a placebo (*n* = 22) and were followed for 10 weeks. In this study, glycemic indices, lipid profiles, anthropometric examinations, blood pressure, high-sensitivity C-reactive protein (hs-CRP), and malondialdehyde (MDA) were measured twice; at baseline and at the end of the study.

**Results:**

After 10 weeks of supplementation, GCE supplementation significantly reduced body weight (*p* = 0.04) and body mass index (BMI) (*p* = 0.03) compared to the placebo. The intention-to-treat (ITT) analysis indicated patients in the GCE group had a lower fasting blood glucose (FBG) concentration compared to the placebo group; however, this decreasing was marginally significant (8.48 ± 8.41 vs. 1.70 ± 5.82 mg/dL, *p* = 0.05). There was no significant difference in insulin levels and HOMA-IR between the groups. At the end of the study, significant changes in systolic blood pressure (SBP) (*p* = 0.01), triglyceride (TG) level (*p* = 0.02), high-density lipoprotein (HDL) (*p* = 0.001), and TG-to-HDL ratio (*p* = 0.001) were found between the intervention and placebo groups. Our trial indicated GCE supplementation had no effect on diastolic blood pressure (DBP), low-density lipoprotein (LDL), or total cholesterol. During the supplementation period, the hs-CRP level significantly decreased in the GCE group compared to the placebo group (*p* = 0.02). No significant changes were observed in the MDA level between the two groups at the end of the study (*p* = 0.54).

**Conclusion:**

Our findings showed beneficial effects of GCE on SBP, TG, hs-CRP, and HDL levels in patients with T2D and overweight/obesity over a 10-week period of supplementation.

**Clinical trial registration:**https://en.irct.ir/trial/48549, identifier [IRCT20090203001640N18].

## Introduction

Generally, patients with type 2 diabetes mellitus (T2D) are at increased risk for various cardiovascular and renal diseases. These patients have several comorbidities, such as fatty liver disease, atherogenic dyslipidemia, and hypertension, which are associated with an increased risk of disability and mortality (1). A total of 425 million adults suffer from diabetes globally, which is anticipated to rise to 629 million by 2040 (2). So, pharmacotherapy and lifestyle changes are necessary to control T2D in the long term (3). Dietary modification is also recommended to reduce the complications of T2D, and currently, most studies have focused on the effects of nutraceuticals on treating or preventing diabetes complications (4).

The green coffee bean is an unroasted coffee fruit that is rich in bioactive phytochemical compounds (5). The main bioactive ingredient of green coffee is chlorogenic acid (CGA), which has beneficial effects on blood pressure, inflammatory markers, oxidative stress, and diabetes in experiments (6). Several mechanisms have been proposed by which CGA exerts its effects, such as inhibiting the cytochrome P450 1A enzymes that increase the pro-inflammatory response (7), increasing the expression of genes that reduce oxidative stress (8), decreasing insulin resistance by activating the hepatic proliferation-activated receptor α (PPAR-α) (9), or improving blood glucose by activating AMP-activated protein kinase (AMPK) (10). However, the health benefits of green coffee for humans are inconsistent. Several randomized controlled trials (RCTs) have evaluated the effects of green coffee extract (GCE) supplementation on cardiometabolic risk factors. Some studies have found that GCE has favorable effects on cardiometabolic risk factors, while others have not (11–15). In addition, most studies have been conducted on healthy adults or subjects with overweight or obesity, and there is a lack of individual studies on patients with T2D (11, 16, 17). Results of a recent meta-analysis on 27 RCTs showed an advantageous influence of GCE on glycemic markers, triglycerides (TG), and high-density lipoprotein (HDL); however, due to the high heterogeneity between studies, the results should be interpreted with caution (18). In regards to inflammatory markers, there are also contradictory results (19).

Despite the protective effects of GCE on chronic diseases (20), research regarding the effects of GCE on T2D is scarce. Moreover, most of the previous studies assessed the effects of CGA rather than GCE in animal models. Besides, findings concerning the effects of GCE are rather inconsistence. Thus, we designed a clinical trial study to assess the effects of GCE supplementation on glycemic indices, lipid profile, hs-CRP, and malondialdehyde (MDA) in patients with T2D and overweight/obesity.

## Methods

### Study design

The present study was a parallel randomized, double-blind, placebo-controlled trial. The National Nutrition and Food Technology Research Institute of Shahid Beheshti University of Medical Sciences’ ethics committee approved this study. It is also registered on the Iranian Registry of Clinical Trials (registration number: IRCT20090203001640N18). At the beginning, the participants signed a “written informed consent form.”

In this study, 44 patients with T2D and overweight/obesity who were referred to the Taleghani Hospital, Tehran, between June 2022 and December 2022 participated. Inclusion criteria were as follows: being aged 30–70 years old, suffering from T2D [fasting blood glucose (FBG) ≥ 126 mg/dL, measured twice, 2-h plasma glucose ≥200 mg/dL, or HbA1C ≥ 6.5%, or taking oral hypoglycemic medicines] (21), having a history of diabetes between 1 and 10 years, and having a body mass index (BMI) of 25–35 kg/m^2^. Exclusion criteria were as follows: receiving insulin therapy, pregnancy or lactation, going on diet during the past 3 months, taking any dietary supplements at least once a week in the past 3 months, and having severe hepatic, renal, and inflammatory diseases. We also removed patients who took less than 80% of their capsules or changed the type or dosage of medications during the intervention. At baseline, patients were asked to complete a questionnaire regarding socio-demographic characteristics, diet and drug histories, and medical history through a comprehensive face-to-face interview.

### Intervention

In this study, the sample size was calculated based on the primary outcome with a power of 80% and an alpha of 0.05. Then, participants were randomly assigned into two groups of 22 patients that received either oral capsules of a GCE or a homologated placebo (starch). Both GCE and placebo capsules were produced by Bonyan Salamat Kasra Co., Tehran, Iran, and were similar in size, shape, and smell. GCE contains 0–2% caffeine and 45–50% CGA by weight. Randomization was conducted using the permuted block method based on sex. Blocked randomization was done with block sizes of four concealed in a container by one of the researchers. The blocks were composed of A and B characters, representing bottles of capsules coded with A or B to ensure concealment. The other investigator randomly assigned the participants to one of the two groups. Subjects were recommended to consume two capsules (each capsule contains 400 mg GCE or placebo) after their main meals to reduce gastrointestinal complications for 10 weeks. All participants were contacted every two weeks to ensure that they complied with the supplementation protocol (adherence of at least 80% was considered good) and to evaluate the side effects. Subjects who reported severe side effects or consumed less than 80% of the capsules were excluded from the study. The participants and researchers were blinded by the intervention. Nutritional recommendations (based on the food guide pyramid) were given to the subjects during the study period, and they were requested to maintain their usual physical activity.

### Dietary assessment

In order to assess dietary changes during the trial period, all the patients were asked to provide two three-day food records (1 weekend day and 2 weekdays) at the beginning and end of the intervention. Food records consisted of the serving size of consumed foods and ingredients. Also, the subjects were interviewed to report their intake based on household measures. The Household Measures and food model booklet with pictures of household items was used to better estimation of portion size of the food and beverages. These measures were used to obtain the grams of food consumed. Nutritionist IV software (First Databank, San Bruno, CA, United States), which is adapted for the national composition food tables, was used to perform nutrient analysis.

### Outcomes assessment

At baseline and at the end of the study, all physical, clinical, and biochemical factors were measured. Weight was measured using a Seca digital scale (Germany) while the subjects were minimally clothed and without shoes, with a precision of 100 g. Also, height was measured using a tape meter attached to the wall to the nearest 0.5 cm. In addition, BMI was calculated by dividing weight (kg) by the square of height (m^2^) at baseline and 10 weeks later. After 10 min of rest, systolic blood pressure (SBP) and diastolic blood pressure (DBP) were measured twice using a digital sphygmomanometer (Citizen, Japan) with a precision of 1 mmHg. Finally, the average of the two measurements was considered the subject’s blood pressure. Physical activity level was assessed using a short form of the International Physical Activity Questionnaire (IPAQ) (22). The physical activity data were reported as metabolic equivalent minutes per week (MET-minutes/wk).

At the beginning and after 10 weeks of intervention, 10 mL of venous blood was drawn from participants after a 12-h fasting period. The blood serum was obtained by centrifugation at a rate of 3,500 rounds per minute. Afterwards, the serum samples were frozen and stored at −70°C until the time of the experiments at the research institute for endocrine sciences, Shaid Beheshti University of Medical Sciences, Tehran, Iran. The serum concentrations of FBG, TG, total cholesterol, and HDL were assessed using an enzymatic colorimetric method (Delta Darman, Iran). Also, the serum insulin levels were measured using the enzyme-linked immunosorbent assay (ELISA) technique (Monobind, Austria). To estimate insulin resistance, the homoeostasis model assessment of insulin resistance (HOMA-IR) was calculated according to the equation by Matthews et al. (23): HOMA-IR = [insulin (Mu/L) × glucose (mg/dL)]/405. The low-density lipoprotein (LDL) levels were calculated using the Friedewald formula: LDL = TC – HDL – (TG/5) (24). The serum hs-CRP levels were assessed using turbidimetry (Delta Darman, Iran). The serum concentration of MDA was measured using a colorimetric method using a commercial kit (Karmania Pars Gene, Iran). The intra-assay coefficients of variation (CVs) for serum FBG, insulin, TG, cholesterol, LDL, HDL, and CRP were 0.92, 6.1, 0.97, 2.8, 1.63, and 7.3, respectively.

### Statistical analysis

The statistical tests were performed using SPSS software (version 20.0; Chicago, IL, United States). The Kolmogorov–Smirnov test was used to test the normality of variable distributions. Quantitative variables are expressed as the mean ± standard deviation (SD), and categorized variables are reported as counts (percent). Baseline characteristics and biochemical variables of the subjects were compared using the student’s *t*-test and the chi-squared test for quantitative and qualitative variables, respectively. A student’s paired *t*-test was used to compare baseline and 10-week values of outcomes within each group. To examine the effect of supplementation on outcomes of interest, an analysis of covariance (ANCOVA) with adjustment for the baseline value, age, sex, smoking, physical activity, medications, and baseline BMI was used. All analyses were conducted using the intention-to-treat (ITT) method. Missing values were replaced by the single imputation method. Two-sided *p* values < 0.05 were considered statistically significant.

## Results

### Participants flow

**Figure 1:**
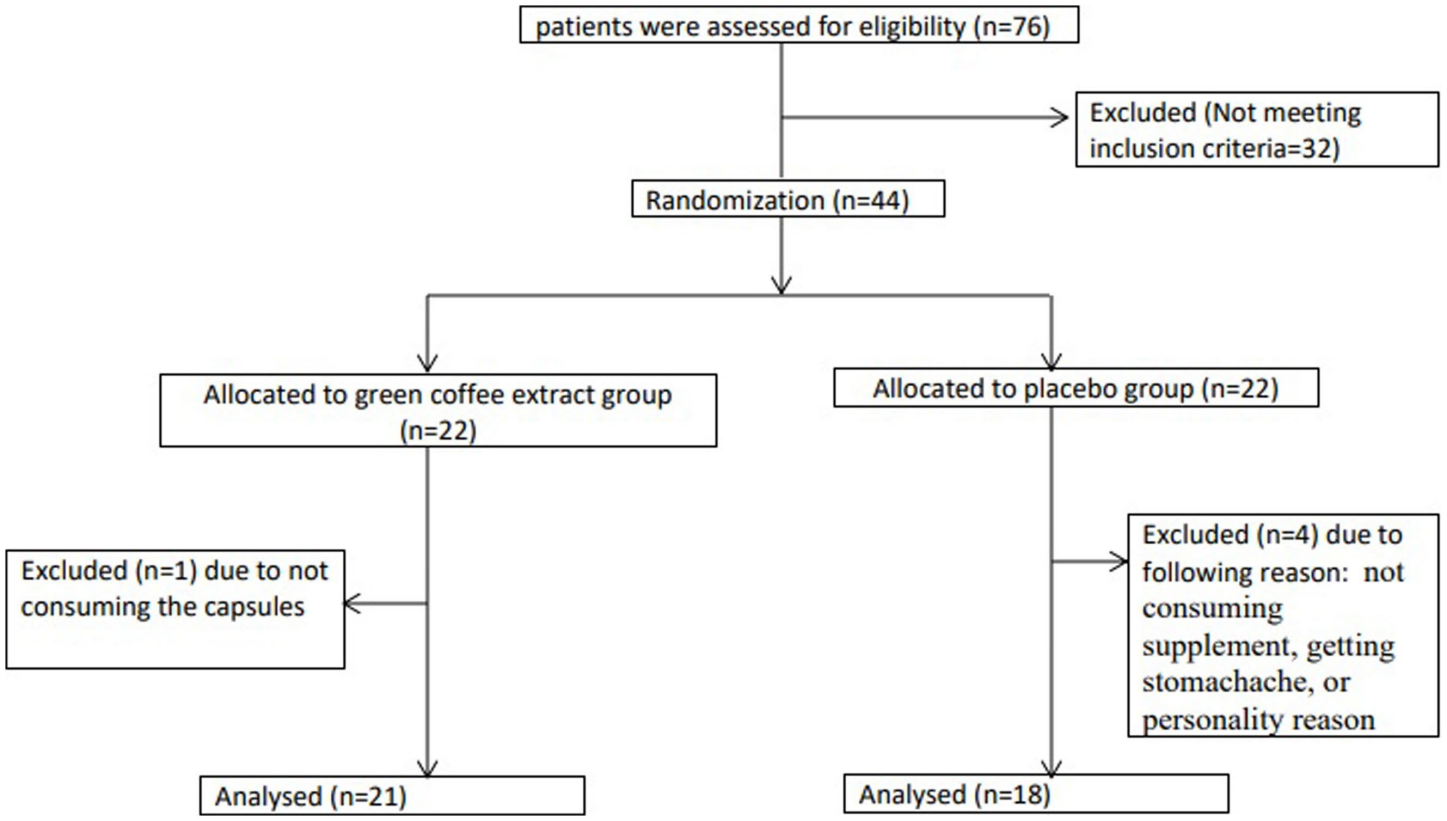
Flow chart of study.

In this RCT, a total of 44 patients with T2D and overweight/obesity were enrolled into two groups: the GCE supplementation group (*n* = 22) and the placebo group (*n* = 22). During the 10-week follow-up, four subjects in the placebo group and one subject in the intervention group dropped out due to poor adherence to the supplementation, side effects such as stomachaches, or being unwilling to continue the study (Figure 1). Finally, 39 subjects completed study, but we analyzed 44 patients using the ITT method. Patients had a compliance rate of 88.6%. We found no major intervention-related adverse effects in either groups during the study.

The baseline characteristics of the participants are shown in Table 1. The mean age of participants was 56.1 ± 6.20 years. Also, 41% of participants were women. No significant differences were found between the GCE and placebo groups in terms of age, BMI, smoking status, or physical activity at baseline.

**Table 1:** Baseline characteristics of the participants.

Variables	Green coffee group (*n* = 22)	Placebo group (*n* = 22)	*p*-value
Age (year)	55.4 ± 6.68	56.7 ± 5.74	0.47
Female (%)	10 (45.5)	8 (36.4)	0.54
Smoking (yes)	6 (27.3)	4 (18.2)	0.47
**Education level (%)**			
Under diploma	16 (72.7)	14 (63.6)	0.51
Diploma and over	6 (27.3)	8 (36.4)	
**Oral drugs (%)**			
Metformin	16 (72.7)	19 (86.4)	0.26
Glybencelamid	6 (27.3)	7 (31.8)	0.74
Statins	6 (27.3)	5 (22.3)	0.72
Physical activity (MET-h/wk)	34.3 ± 3.95	35.3 ± 3.64	0.41

Data are presented as percent and mean ± standard deviation. *p* value is reported based on independent *t*-test for continuous variables and chi-square for non-continuous variable.

The dietary intake of participants is shown in Table 2. No significant differences were observed in the dietary intake between the GCE and placebo groups at the baseline of the trial. Also, no significant changes were observed in the dietary intakes between the two groups after 10 weeks, except for the percent of energy from saturated fatty acid (SFA) consumption. After 10 weeks, subjects in the placebo group consumed less SFA compared to the GCE group (8.22 ± 1.85 vs. 9.55 ± 1.54% of energy).

**Table 2:** Dietary intake of participants at the baseline and after 10 weeks supplementation.

Dietary factors	Time	Green coffee group (*n* = 22)	Placebo group (*n* = 22)	*p*-value^a^
Energy (kcal/d)	Baseline	2,266 ± 232	2,186 ± 174	0.20
	10 weeks	2,123 ± 243	2,109 ± 220	0.19
Carbohydrate (% of energy)	Baseline	61.3 ± 4.55	58.2 ± 7.52	0.11
	10 weeks	58.1 ± 3.81	59.9 ± 5.74	0.28
Protein (% of energy)	Baseline	15.3 ± 1.61	14.9 ± 1.71	0.38
	10 weeks	15.1 ± 2.71	15.7 ± 1.68	0.65
Fat (% of energy)	Baseline	26.8 ± 4.77	30.3 ± 6.90	0.07
	10 weeks	29.6 ± 5.44	27.5 ± 5.45	0.21
SFA (% of energy)	Baseline	8.99 ± 2.93	9.58 ± 2.85	0.50
	10 weeks	9.55 ± 1.54	8.22 ± 1.85	0.009
MUFA (% of energy)	Baseline	9.91 ± 2.08	9.45 ± 2.48	0.51
	10 weeks	10.3 ± 2.67	9.42 ± 2.31	0.21
PUFA (% of energy)	Baseline	6.25 ± 2.09	5.67 ± 1.60	0.30
	10 weeks	6.06 ± 2.43	6.15 ± 2.21	0.99
Fiber (g/d)	Baseline	41.3 ± 12.07	39.1 ± 6.01	0.44
	10 weeks	40.5 ± 12.3	39.3 ± 8.23	0.65
Cholesterol (mg/d)	Baseline	200 ± 84.7	188 ± 66.9	0.59
	10 weeks	198 ± 72.8	182 ± 86.5	0.43
Sodium (mg/d)	Baseline	3,256 ± 1,021	3,358 ± 1,113	0.75
	10 weeks	3,342 ± 1,284	2,885 ± 633	0.09
Magnesium (mg/d)	Baseline	483 ± 95.9	446 ± 75.9	0.16
	10 weeks	434 ± 98.1	455 ± 77.6	0.41
Potassium (mg/d)	Baseline	4,534 ± 1,045	4,373 ± 840	0.57
	10 weeks	4,183 ± 1,120	795 ± 193	0.93
Calcium (mg/d)	Baseline	1,395 ± 402	1,331 ± 284	0.54
	10 weeks	1,318 ± 512	1,245 ± 279	0.60
Selenium (μg/d)	Baseline	111 ± 24.2	121 ± 29.6	0.22
	10 weeks	105 ± 6.1	117 ± 6.7	0.18
Vitamin C (mg/d)	Baseline	173 ± 55.4	146 ± 66.3	0.15
	10 weeks	134 ± 12.2	142 ± 13.5	0.67
Vitamin E (mg/d)	Baseline	10.3 ± 2.9	12.6 ± 7.7	0.20
	10 weeks	10.6 ± 0.8	10.1 ± 0.8	0.68

Data are presented as mean ± standard deviation.

a *p* value is reported based on independent *t*-test for baseline values and ANCOVA test for final values.

MUFA, monounsaturated fatty acids; PUFA, polyunsaturated fatty acids; SFA, saturated fatty acids.

### Anthropometric and glycemic induces

Anthropometric and glycemic induces at baseline and after 10 weeks of supplementation are reported in Table 3. Body weight (*p* = 0.04) and BMI (*p* = 0.03) significantly decreased in the GCE group compared to the placebo group after 10 weeks of supplementation. The ITT analysis indicated patients in the GCE group had a lower FBG concentration compared to the placebo group; however, this decreasing was marginally significant (8.48 ± 8.41 vs. 1.70 ± 5.82 mg/dL, *p* = 0.05) than the placebo group. During the intervention, no significant difference in insulin level (−6.23 ± 13.2 vs. −3.59 ± 6.28 μIU/dL, *p* = 0.20) and HOMA-IR (−2.84 ± 5.72 vs. −1.07 ± 2.46, *p* = 0.08) was found between the two groups.

**Table 3:** Anthropometric and glycemic indices at baseline and after 10 weeks supplementation.

Variable	Time	Green coffee group (*n* = 22)	Placebo group (*n* = 22)	*p*-value^a^
Weight (kg)	Baseline	75.8 ± 4.49	73.7 ± 4.40	0.04
	10 weeks	73.3 ± 3.50^c^	72.9 ± 3.75	
	Mean changes	−2.62 ± 2.23	−0.72 ± 2.13	
BMI (kg/m^2^)	Baseline	27.9 ± 1.39	27.4 ± 1.25	0.03
	10 weeks	26.9 ± 1.37^c^	27.0 ± 1.15	
	Mean changes	−1.01 ± 0.94	−0.3 ± 0.83	
FBG (mg/dL)	Baseline	141 ± 18.4	141 ± 12.9	0.05
	10 weeks	132 ± 13.5^c^	138 ± 11.8	
	Mean changes	−8.48 ± 8.41	−1.70 ± 5.82	
Insulin (μIU/dL)	Baseline	17.7 ± 18.9	16.2 ± 9.27	0.20
	10 weeks	11.5 ± 8.2^b^	12.6 ± 4.12^b^	
	Mean changes	−6.23 ± 13.2	−3.59 ± 6.28	
HOMA-IR	Baseline	6.76 ± 7.89	5.47 ± 3.98	0.08
	10 weeks	3.92 ± 2.97^b^	4.40 ± 1.80	
	Mean changes	−2.84 ± 5.72	−1.07 ± 2.46	

Data are presented as mean ± standard deviation.

a *p* value is reported based on ANCOVA test with adjustment for baseline values, age, sex, smoking, physical activity, medications, and baseline BMI.

b *p* value is reported based on paired *t*-test (*p* < 0.05).

c *p* value is reported based on paired *t*-test (*p* < 0.01).

BMI, body mass index; FBG, fasting blood glucose; HOMA-IR, homeostatic model assessment for insulin resistance.

### Blood pressure and lipid profile

Blood pressure and lipid profile at baseline and after 10 weeks of supplementation are reported in Table 4. At the end of the study, GCE significantly reduced SBP (−5.56 ± 3.41 vs. −0.90 ± 2.67 mmHg, *p* = 0.01), TG level (−49.7 ± 72.5 vs. −4.40 ± 98.6 mg/dL, *p* = 0.02), and TG-to-HDL ratio (−1.40 ± 1.83 vs. 0.13 ± 2.12, *p* = 0.001), and increased HDL level (3.22 ± 5.10 vs. 1.73 ± 6.10 mg/dL, *p* = 0.001) compared to the placebo. Our trial indicated no effect of GCE on DBP, LDL, or total cholesterol after 10 weeks of supplementation.

**Table 4:** Blood pressure and lipid profile at baseline and after 10 weeks supplementation.

Variable	Time	Green coffee group (*n* = 22)	Placebo group (*n* = 22)	*p*-value^a^
SBP (mmHg)	Baseline	130 ± 6.27	124 ± 5.67^b^	0.01
	10 weeks	125 ± 4.31^d^	123 ± 4.41	
	Mean changes	−5.56 ± 3.41	−0.90 ± 2.67	
DBP (mmHg)	Baseline	80.5 ± 6.37	78.4 ± 5.25	0.51
	10 weeks	80.2 ± 5.13	77.8 ± 4.35	
	Mean changes	−0.33 ± 2.77	−0.61 ± 2.58	
Triglyceride (mg/dL)	Baseline	187 ± 86.4	172 ± 109	0.02
	10 weeks	138 ± 45.9 ^d^	168 ± 47.4	
	Mean changes	−49.7 ± 72.5	−4.40 ± 98.6	
Cholesterol (mg/dL)	Baseline	176 ± 36.2	172 ± 40.9	0.34
	10 weeks	173 ± 42.2	16 ± 45.5	
	Mean changes	−3.93 ± 29.9	−10.6 ± 28.0	
LDL (mg/dL)	Baseline	171 ± 34.4	172 ± 40.9	0.50
	10 weeks	154 ± 35.1^c^	163 ± 45.6	
	Mean changes	−17.1 ± 28.2	−9.78 ± 33.8	
HDL (mg/dL)	Baseline	42.5 ± 11.8	40.7 ± 7.83	0.001
	10 weeks	45.7 ± 12.1^d^	39.0 ± 5.39	
	Mean changes	3.22 ± 5.10	−1.73 ± 6.10	
TG/HDL ratio	Baseline	4.64 ± 2.31	4.29 ± 2.32	0.001
	10 weeks	3.23 ± 1.34^d^	4.42 ± 1.44	
	Mean changes	−1.40 ± 1.83	0.13 ± 2.12	

Data are presented as mean ± standard deviation.

a *p* value is reported based on ANCOVA test with adjustment for baseline values, age, sex, smoking, physical activity, medications, and baseline BMI.

b *p* value is reported based on independent *t*-test (*p* < 0.05).

c *p* value is reported based on paired *t*-test (*p* < 0.05).

d *p* value is reported based on paired *t*-test (*p* < 0.01).

BMI, body mass index; DBP, diastolic blood pressure; HDL, high density lipoprotein; LDL, low density lipoprotein; SBP, systolic blood pressure.

### Hs-CRP and MDA levels

Table 5 shows hs-CRP and MDA concentrations at baseline and end of the study in the GCE and placebo groups. During the supplementation period, the hs-CRP level significantly decreased in the GCE group compared to the placebo group (*p* = 0.02). No significant changes were observed in the MDA level between the two groups at the end of the study.

**Table 5:** CRP and MDA at baseline and after 10 weeks supplementation.

Variable		Green coffee group (*n* = 22)	Placebo group (*n* = 22)	*p*-value^a^
CRP (mg/L)	Baseline	3.31 ± 2.41	2.69 ± 2.13	0.02
	10 weeks	2.28 ± 1.46^b^	2.88 ± 1.84	
	Mean changes	−1.04 ± 1.21	0.18 ± 1.85	
MDA (μmol/L)	Baseline	1.44 ± 0.96	1.15 ± 0.59	0.18
	10 weeks	1.19 ± 0.78	1.25 ± 0.59	
	Mean changes	−0.25 ± 1.06	0.09 ± 0.67	

Data are presented as mean ± standard deviation.

a *p* value is reported based on ANCOVA test with adjustment for baseline values, age, sex, smoking, physical activity, medications, and baseline BMI.

b *p* value is reported based on paired *t*-test (*p* < 0.01).

BMI, body mass index; CRP, C-reactive protein; MDA, malondialdehyde.

## Discussion

In this RCT among participants with T2D and overweight/obesity, GCE supplementation with doses of 800 mg/d for 10 weeks led to a significant decrease in weight, BMI, SBP, HOMA-IR, serum TG, and CRP levels, and a significant increase in serum HDL levels. Our results did not show beneficial effects of GCE supplementation on SBP and serum concentrations of insulin, total cholesterol, LDL, and MDA. Evidence has verified the safety of the dose and duration of the GCE intervention. According to the previous studies, which used a dosage of 400–1,000 mg/d with a duration of 8–12 weeks, we chose this dose and duration in the current study (25).

### Anthropometric and lipid profile

Our results regarding body weight and BMI were in line with findings from previous studies (12, 26). In a trial of the effect of GCE on body composition in women with obesity, GCE supplementation (400 mg/d for 8 weeks) reduced body weight and BMI (12). Also, a meta-analysis has reported that consumption of GCE with a dosage of 180–200 mg/d can lead to a weight loss of 2.5 kg. However, there was a high heterogeneity (*I*^2^ = 97%) between studies that made conclusions with difficulty (26) Green coffee is a rich source of a natural antioxidant known as CGA. Therefore, the weight-lowering effect of green coffee may be due to the CGA (27). CGA can contribute to body weight loss through its anti-hyperlipidemia effects. In an animal model, CGA can reduce cholesterol synthesis and increase fatty acid oxidation by inhibiting β-hydroxy-β-methyl glutaric acyl-coenzyme A reductase (28). These mechanisms can explain the reduced effects of GCE supplementation on lipid profile in this trial. Also, CGA has a reducing effect on the deposition of fatty acids in the adipose tissue by decreasing the serum insulin level, which leads to body weight loss (12). In the current trial, GCE consumption caused a significant reduction and increase in TG and HDL levels, respectively. A trial on subjects with obesity who consumed 1,000 mg/d of green coffee for 6 weeks reported a significant improvement in their lipid profile (15). Similar to our findings, in a RCT on patients with nonalcoholic fatty liver disease (NAFLD) who consumed 400 mg of GCE for 8 weeks, they observed a significant increase in HDL levels (16). But this trial did not detect any significant effect of GCE on TG, LDL, or total cholesterol compared to the placebo. However, in another trial on subjects with metabolic syndrome, consumption of 400 mg GCE for 8 weeks had no improvement effect on lipid profile parameters (11). Several mechanisms were proposed for the anti-lipogenic effect of CGA. This phytochemical component has an inhibitory effect on pancreatic lipase activity, which decreases fat absorption. In addition, CGA has an increasing and decreasing effect on fatty acid oxidation and fatty acid biosynthesis, respectively (8, 29, 30).

### Glycemic indices

Our trial showed that GCE could decrease FBG compared to the placebo; however this reduction was marginally significant. But this study did not detect any significant effect of GCE on insulin and HOMA-IR. Some of previous studies have reported different results (11, 31). Roshan et al. (11) found that the subjects in the GCE group (dosage of 800 mg/d and duration of 8 weeks) had significantly greater decreases in blood glucose and insulin resistance compared to the placebo. In an animal study, GCE treatment effectively reduced the FBG (dosage of 100 mg/kg and duration of 6 weeks) compared to the placebo group in high-fat diet (HFD)-induced obese mice (31). Also, in another animal study, GCE (80 mg/d) resulted in attenuation of HFD-induced insulin resistance after 14 weeks (32). But, in line with our findings an HFD diet plus 0.5% w/w GCE in mice with metabolic syndrome did not improve insulin resistance after 12 weeks (33). A meta-analysis that summarized the results from six interventional studies reported that GCE has a reducing effect on blood glucose (34). Also, this meta-analysis concluded that GCE has no effect on insulin or HOMA-IR (34). Some reason can explain these slight contradictory results; the difference in population study can be one of the reasons for the inconsistency in the results. In current study, we assessed the effects of GCE on diabetic patients, but previous studies assessed the effects of GCE on different population. Also, the difference in the baseline FBG and insulin levels can be another reason for the inconsistency in the results. It is suggested that CGA could improve FBG by activating AMP-activated protein kinase (AMPK), and activation of this kinase consequently increased glucose uptake in the cells by glucose transporter 4 (GLU4) fusion with the plasma membrane (10). Also, CGA can inhibit the key enzymes linked to the absorption of glucose (including pancreatic amylase isoenzymes I and II, α-glucosidase, and α-amylase), leading to postprandial blood glucose improvement (10). Furthermore, CGA can decrease glucose production (glycogenolysis and gluconeogenesis) by inhibiting the glucose-6-phosphatase enzyme (35). It is thought that GCE lowers insulin resistance by the activation of insulin receptor substrate-1 via inhibiting c-Jun N-terminal kinase phosphorylation. This causes GLUT4 translocation to the adipocyte membrane (36). Also, GCA can decrease insulin resistance by activating the hepatic proliferation-activated receptor α (PPAR-α), which facilitates the clearing of lipids from the liver (9).

### Hs-CRP and MDA levels

Our trial demonstrated that GCE administration for 10 weeks in patients with T2D significantly decreased CRP. Our results were in agreement with a double-blind, placebo-controlled, randomized trial of the effect of GCE on adult patients with non-alcoholic fatty liver disease conducted by Shahmohammadi et al. (17). They reported that GCE supplementation has an improving effect on hs-CRP levels. But, in a placebo-controlled double-blind pilot study on healthy men, GCE had no effect on CRP compared to the placebo group (14). The inconsistent results can be due to differences in the duration and dosage of the intervention, sample size, and population. The main reason for this inconsistency in the result can be due to the difference in population study. In comparison with the previous study that has assessed the effects Of GCE on healthy subjects, but we assessed the effects of GCE on diabetic population, that these subjects have more inflammation than healthy subjects. This study was conducted only on men subjects, which can be another reason of inconsistency in the results. Also, all subjects in the current study have higher BMI and age compared to the subjects of previous study. A recent meta-analysis of eight trials found that GCE supplementation (dosage 50–1,200 mg/d, duration of 8–12 weeks) has no effect on CRP (19). However, based on the results of this meta-analysis, GCE supplementation has a decreasing effect on tumor necrosis factor alpha (TNF-α) as an inflammatory biomarker (37). CGA, by increasing the expression of genes encoding enzymes, including glutathione peroxidase and superoxide dismutase, exerts reducing effects on oxidative stress (8). Also, CGA has improved effects on inflammation by inhibiting the cytochrome P450 1A enzymes that increase the pro-inflammatory response in peripheral blood mononuclear cells (7). Our findings did not show a significant effect of GCE on MDA levels after 10 weeks.

### Study strengths and limitations

The randomized, placebo-controlled design was the main strength of our study. However, using a fixed dose of GCE and a short period of intervention were the limitations of the current study. Therefore, additional studies with a large sample size, different dosages, and longer durations are required to demonstrate the potential effects of GCE in patients with T2D.

## Conclusion

Based on the findings of our study, GCE administration at a dosage of 800 mg/d for 10 weeks in patients with T2D decreased SBP, TG, and CRP and increased HDL compared to placebo. Moreover, FBG reduction in GCE group was marginally significant compared to the placebo group. Therefore, GCE may have a beneficial effect on lipid profile and inflammation in patients with T2D.

## Data availability statement

The original contributions presented in the study are included in the article/supplementary material, further inquiries can be directed to the corresponding authors.

## Ethics statement

The studies involving humans were approved by Faculty of Nutrition and Food Technology, National Nutrition and Food Technology, Research Institute, Shahid Beheshti University of Medical Sciences, Tehran, Iran. The studies were conducted in accordance with the local legislation and institutional requirements. The participants provided their written informed consent to participate in this study.

## Author contributions

SK-M and PM conceived and designed the study. SK-M, PM, and MH contribute to data collection. SK-M and MG analyzed the data and drafted the initial manuscript. All authors contributed to the article and approved the final manuscript.
